# Phytoplankton Community Structure Is Driven by Stratification in the Oligotrophic Mediterranean Sea

**DOI:** 10.3389/fmicb.2019.01698

**Published:** 2019-07-24

**Authors:** Catalina Mena, Patricia Reglero, Manuel Hidalgo, Eva Sintes, Rocío Santiago, Melissa Martín, Gabriel Moyà, Rosa Balbín

**Affiliations:** ^1^Ecosystem Oceanography Group (GRECO), Instituto Español de Oceanografía, Centre Oceanogràfic de les Balears, Palma, Spain; ^2^Department of Limnology and Bio-Oceanography, University of Vienna, Vienna, Austria; ^3^Departament de Biologia, Universitat de les Illes Balears, Palma, Spain

**Keywords:** phytoplankton size structure, picoeukaryotes, stratification, mesoscale, oligotrophy, carbon biomass, Mediterranean Sea

## Abstract

The phytoplankton community composition, structure, and biomass were investigated under stratified and oligotrophic conditions during summer for three consecutive years in the Mediterranean Sea. Our results reveal that the phytoplankton community structure was strongly influenced by vertical stratification. The thermocline separated two different phytoplankton communities in the two layers of the euphotic zone, characterized by different nutrient and light availability. Picoplankton dominated in terms of abundance and biomass at all the stations sampled and throughout the photic zone. However, the structure of the picoplanktonic community changed with depth, with *Synechococcus* and heterotrophic prokaryotes dominating in surface waters down to the base of the thermocline, and *Prochlorococcus* and picoeukaryotes contributing relatively more to the community in the deep chlorophyll maximum (DCM). Light and nutrient availability also influenced the communities at the DCM layer. *Prochlorococcus* prevailed in deeper DCM waters characterized by lower light intensities and higher picophytoplankton abundance was related to lower nutrient concentrations at the DCM. Picoeukaryotes were the major phytoplankton contributors to carbon biomass at surface (up to 80%) and at DCM (more than 40%). Besides, contrarily to the other phytoplankton groups, picoeukaryotes cell size progressively decreased with depth. Our research shows that stratification is a major factor determining the phytoplankton community structure; and underlines the role that picoeukaryotes might play in the carbon flux through the marine food web, with implications for the community metabolism and carbon fate in the ecosystem.

## Introduction

Oligotrophic oceanic regions comprise vast areas of the global ocean. These regions are predicted to expand following the strengthening of the water column stratification with increasing temperatures (Polovina et al., 2008; Gruber, 2011; Capotondi et al., 2012), resulting in reduced nutrient fluxes and primary productivity (Falkowski and Oliver, 2007; Lozier et al., 2011). Oligotrophic areas with intermittent or irregular nutrient pulses at a regional scale are ideal to study phytoplankton dynamics as shifts in community structure are promoted (Siokou-Frangou et al., 2010). Yet, given the relevance of phytoplankton modulating the marine food webs and carbon fluxes, the impact of phytoplankton to the regional ecology, particularly in intermittent oligotrophic areas, remains largely unknown.

Phytoplankton size is considered to be one of the most important functional traits influencing various biological and ecological processes (Marañón, 2015; Acevedo-Trejos et al., 2018; Villarino et al., 2018). Cell size affects nutrient uptake, i.e., smaller cells have an advantage over larger cells for nutrient acquisition in nutrient-depleted environments as a consequence of their higher surface-area-to-volume ratio (Marañón et al., 2012). Sinking and grazing rates are also size-dependent as larger cells sink more efficiently (Mackinson et al., 2015) and faster (Marañón, 2015) than smaller phytoplankton cells. It has been argued that larger phytoplankton cells can escape grazing more easily than smaller cells due to the different generation times of their main predators, metazoans and protists, respectively (Acevedo-Trejos et al., 2015; Marañón, 2015 and references therein). Therefore, the size structure of the phytoplankton community is a critical issue to foresee future carbon cycling and trophic regimes, since it determines the carbon fate in the ecosystem toward higher trophic levels, export to the deep ocean or remineralization within the photic zone (Behrenfeld et al., 2015; Irwin et al., 2015).

The Mediterranean Sea is a semi-enclosed oligotrophic sea, characterized by complex physical and biological dynamics, strong seasonality (D’Ortenzio and Ribera d’Alcalà, 2009; Christaki et al., 2011) and regional structuring of biophysical processes (Rossi et al., 2014; Basterretxea et al., 2018). The intermittent nutrient enrichment is an important factor controlling Mediterranean Sea regional production, affecting the size structure of the phytoplankton and promoting the switch between microbial and classical food web (Siokou-Frangou et al., 2010). The Balearic Sea region is a highly dynamic region characterized by the presence of a salinity front and other diverse mesoscale structures such as eddies or filaments during summer (Millot, 1999; Balbín et al., 2014). Regional phytoplankton biomass and productivity are known to be highly influenced by mesoscale processes, since they strongly affect resource availability (Fiala et al., 1994; Basterretxea and Arístegui, 2000; Jacquet et al., 2002; Landry, 2002; Brown et al., 2008; Cotti-Rausch et al., 2016). In addition, the Balearic Sea region during summer is characterized by intense stratification and nutrient depletion. During this season, the water column stratification results in a two-layered euphotic zone (Coale and Bruland, 1987), with the upper layer being nutrient limited and the lower layer being light limited (Mignot et al., 2014). Regardless of the general oligotrophic condition in this region, many studies have described a surprising diversity and biomass of higher trophic level communities (Alemany et al., 2006; Rodriguez et al., 2013; Fernández de Puelles et al., 2014; Hidalgo et al., 2014; Laiz-Carrión et al., 2015; Reglero et al., 2017), often utilizing chlorophyll-*a* (Chl-*a*) or fluorescence estimates as a proxy for primary production biomass to assess food web linkages of many marine species. However, the relation between carbon biomass (C) and Chl-*a* concentration (C:Chl-*a*) in phytoplankton cells is largely variable and is influenced by light, nutrients and temperature (Jakobsen and Markager, 2016). Moreover, when available, the estimation of this ratio is biased by potential errors both in Chl-*a* and carbon measurements (Jakobsen and Markager, 2016). Thus, this raises the need to obtain a more comprehensive knowledge of the phytoplankton community dynamics in the area to better understand the trophic links and ecosystem functioning.

The aim of this study was to identify the mechanisms driving the phytoplankton community composition and size structure in relation to vertical stratification and mesoscale dynamics in this non-steady state oligotrophic region. We explored whether the two-layered euphotic zone holds two separated communities and whether these communities are influenced by the mesoscale front structure. Here, we hypothesize that vertically the community structure is strongly influenced by the stratification due to bottom-up control, however, the horizontal patterns may be driven by other factors related to the temporal and varying mesoscale dynamics.

## Materials and Methods

### Study Area and Sampling

**FIGURE 1:**
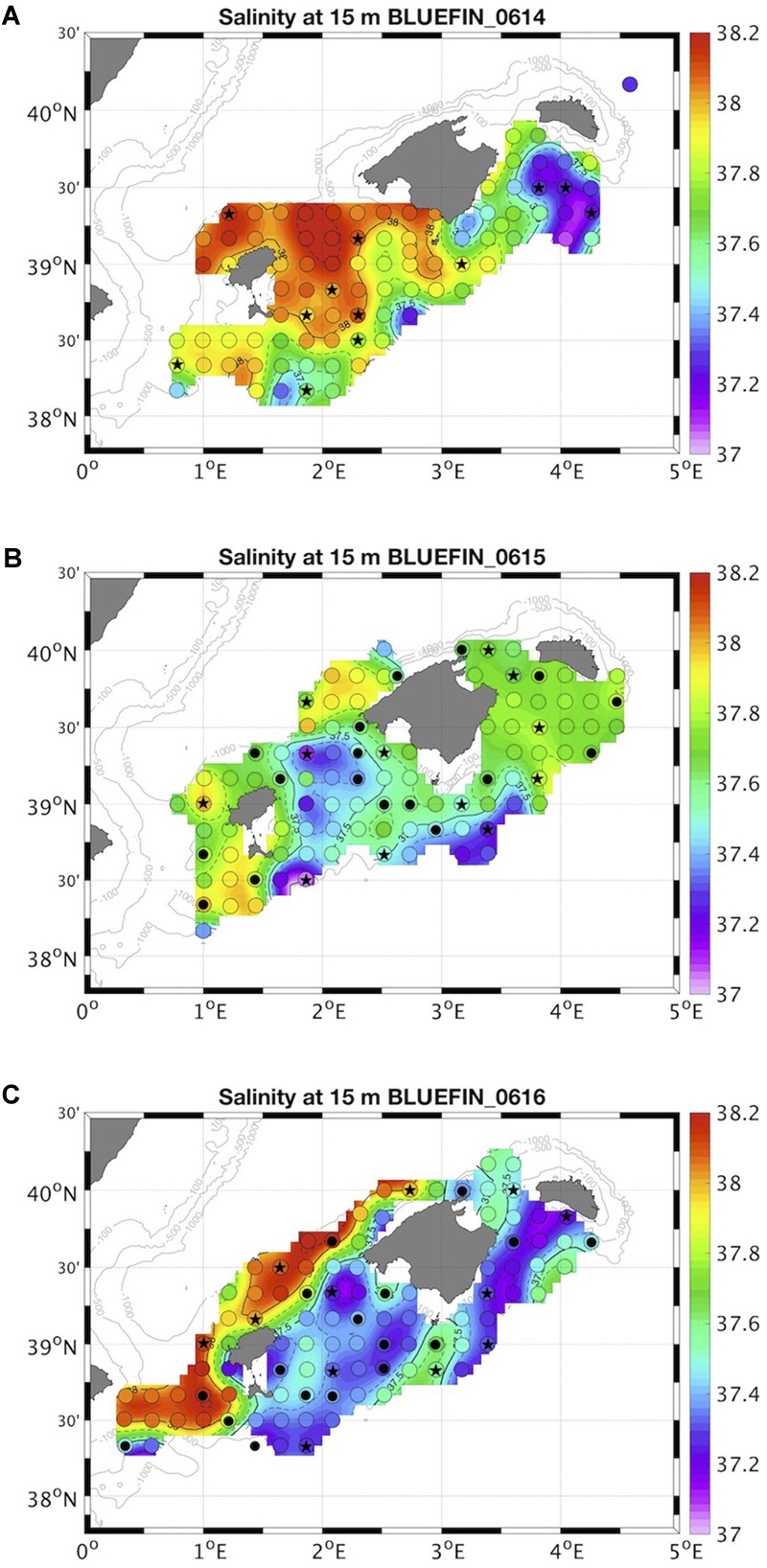
Location of sampled stations in **(A)** 2014, **(B)** 2015, and **(C)** 2016 superimposed to the interpolated surface salinity, following a systematic survey design (open circles) (Alemany et al., 2010). Salinity around 37.5 indicate the front location at surface. Picoplankton was sampled from surface to 200 m depth at all stations (dots and stars). Nano- and microphytoplankton were sampled at surface and DCM at the stations marked with star.

The study was carried out in the Balearic Sea region, western Mediterranean, during three *BLUEFIN* cruises (June–July 2014, 2015, and 2016) on board the R/V *SOCIB*. Sampling was designed to investigate a salinity front formed every summer due to the convergence between newly arrived Atlantic water (AW), moving northward, and the resident AW (Balbín et al., 2014; Figure 1). Samples were collected with Niskin bottles mounted in a CTD-rosette system at a total of 69 stations (12, 29, and 28 stations in 2014, 2015, and 2016, respectively). Samples for abundance and biomass characterization of all phytoplankton size fractions were collected at surface and deep chlorophyll maximum (DCM). In addition, samples to characterize the picoplankton fraction, Chl-*a* and inorganic nutrients concentration were collected at 7 depths: surface, 25, 50, DCM, 75, 100, and 200 m. The fluorescence profile during the CTD downcast was used to estimate the depth of the DCM.

Temperature and salinity were recorded using a SBE911 CTD sensor. Oxygen concentration was measured using a SBE43 sensor mounted on the CTD and calibrated using the Winkler method (Strickland and Parsons, 1968). Apparent oxygen utilization (AOU) was calculated as the difference between the oxygen solubility values at saturation with respect to the atmosphere and the measured dissolved oxygen concentration (computed in Ocean Data View software; Schlitzer, 2006). Photosynthetically active radiance (PAR) and fluorescence were measured using a Biospherical and SeaPoint Fluorometer sensors, respectively. Hydrographic parameters were processed using the standard Sea-Bird Electronics methods. The mixed layer (ML) depth was calculated as the depth where the density sigma was (S, T) = (Sref, Tref-deltaT), being S, T salinity and temperature, respectively, whereas Sref and Tref are salinity and temperature at 10 m depth and deltaT is equal to 0.5°C. The vertical stratification index (VSI) was estimated to characterize the degree of vertical stratification of the water column, calculated using the formula: **Σ** |σ𝜃(m + 1) – σ𝜃(m)|, where σ𝜃 is the potential density anomaly and *m* is the depth in meters ranging from 5 to 80 m.

### Inorganic Nutrients Concentration

Samples for dissolved inorganic nutrients were collected in 12 mL high-density polyethylene vials and stored frozen at -20°C until processing at the home laboratory. Nitrate and nitrite (NO_3_^-^ + NO_2_^-^, hereafter nitrogen), phosphate (PO_4_^3-^), and silicate (SiO_4_^2-^) concentrations were determined using a QuAAtro Gas Segmented Continuous Flow Analyser (SEAL Analytical) following colorimetric protocols (Murphy and Riley, 1962; Strickland and Parsons, 1968; Grasshoff et al., 1983). Detection limits for the procedures were 0.014, 0.007, and 0.032 μM for NO_3_^-^ + NO_2_^-^, PO_4_^3-^, and SiO_4_^2-^, respectively. Nitrogen-to-phosphorous (N:P) ratio was calculated by dividing nitrogen concentration (NO_3_^-^ + NO_2_^-^) by phosphate concentration.

### Chlorophyll-*a* Concentration

Seawater samples (1 L) for total Chl-*a* concentration were filtered through GF/F Whatman glass fiber filters and stored at -20°C. Additionally, seawater (1 L) was filtered onto 20 and 2 μm Nucleopore polycarbonate filters to evaluate size-fractionated Chl-*a* during *BLUEFIN*-2016 cruise. All filters were stored at -20°C until further processing at the home lab. Total and size-fractionated Chl-*a* were extracted in cold acetone (90%) for 24 h and analyzed using a Turner-Designs 10AU Fluorometer following Holm-Hansen et al. (1965) method. Picoplankton and nanoplankton Chl-*a* fractions were calculated by subtracting the 2 μm Chl-*a* from the total Chl-*a* concentration and the 20 μm Chl-*a* from the 2 μm Chl-*a* concentration, respectively. Microplankton Chl-*a* fraction corresponded to the 20 μm Chl-*a* concentration.

### Phytoplankton and Heterotrophic Prokaryotes Abundance

Duplicate samples (1.5 mL) of seawater were fixed with glutaraldehyde (0.1% final concentration) for 10 min, frozen in liquid nitrogen and stored at -80°C until analysis to estimate picoplankton (0.2–2 μm) abundance. Picophytoplankton cells (*Prochlorococcus, Synechococcus*, and picoeukaryotes) were enumerated on a FACSAria II flow cytometer (BD Biosciences) based on their side scatter (SSC) versus red fluorescence and orange versus red fluorescence cytogram (Supplementary Figures S1A,B; Marie et al., 2000). Each group was delimited in the cytogram plot by drawing a gate using the BD FACSDiva Software, adjusting the gating settings for each sample. Flow rate calibration was performed daily. Prior to analysis, samples to enumerate total prokaryotes were stained with SYBR Green I (Sigma-Aldrich) at 1x final concentration for 10 min in the dark. Subsequently, prokaryotes were counted on an ACCURI C6 flow cytometer (BD Biosciences) based on their SSC versus green fluorescence signals (Supplementary Figure S1C; Brussaard, 2004). Gating was manually adjusted for every sample. Fluorescent beads (Fluospheres polystyrene 1.0 μm, Molecular probes) were added to each sample for both picophytoplankton and total prokaryotes analysis as an internal standard. All fluorescence and scattering signals were recorded and normalized to the beads signals. Heterotrophic prokaryotes abundance was calculated by subtracting *Prochlorococcus* and *Synechococcus* from total prokaryotes counts.

Seawater samples for nano- (2–20 μm) and microphytoplankton (>20 μm) enumeration (250 mL) were collected at 12 stations on each cruise (Figure 1) at two depths: surface and DCM. The samples were preserved with Lugol’s iodine solution and stored in the dark until analysis. Nano- and microphytoplankton were counted after sedimentation of the lugol-preserved sample for 48–72 h following the Utermöhl method (Utermöhl, 1958) using a Zeiss Axiovert inverted microscope. Nanophytoplakton was counted at a magnification of 400x in random fields until at least 100 cells of the most abundant group were counted. Microphytoplankton counts were performed at a magnification of 100x examining the entire sedimentation chamber. Phytoplankton cells were identified at the lowest achievable taxonomic level. However, two major taxonomic groups were considered for community analyses purposes, diatoms and dinoflagellates, due to the difficulty of identifying organisms at lower taxonomical level. The nanophytoplankton cells were separated into two size groups: small (2–5 μm) and large (5–20 μm) nanophytoplankton, the latter including diatoms and dinoflagellates smaller than 20 μm.

### Phytoplankton Carbon Biomass Estimates

Carbon biomass was estimated using volume-to-carbon conversion factors. In order to estimate picophytoplankton cell volume, an empirical calibration was performed between the forward scatter signal (FSC) obtained by flow cytometry and the cell volume determined by epifluorescence microscopy on the same samples. The FSC signal of the different phytoplankton populations was normalized by the beads fluorescence signal for every sample. Twenty one samples from different stations and depths were selected for the calibration. Water samples (50 mL) were filtered onto 0.2 μm polycarbonate filters for epifluorescence analysis. *Synechococcus* and picoeukaryotic cells were identified based on their autofluorescence using blue excitation filter set in a Leica DM2500 microscope. Mean cell size was estimated based on a total of 3200 and 1000 different *Synechococcus* and picoeukaryotic cells, respectively. For volume calculation, a spherical shape was assumed for all groups. Then, the linear regression model between the calculated cell volume and the normalized FSC value (FSC_n_) of these two groups for each sample was used to estimate cell volumes of the three picophytoplankton groups distinguished, i.e., including *Prochlorococcus* (Cell volume = 1.01 + 1.61 log(FSC_n_), *R*^2^ = 0.943). Subsequently, picophytoplankton cell volumes were converted to carbon by applying the following conversion factors: 240, 230, and 237 pg C μm^-3^ for *Prochlorococcus, Synechococcus*, and picoeukaryotes (Worden et al., 2004), respectively. Nano- and microphytoplankton cell volumes were calculated attributing specific geometric shapes to different genera or group following Vadrucci et al. (2007), using average cell dimensions measured in all samples by microscopy. The same conversion factor as for picoeukaryotes was used for nanophytoplankton. Microphytoplankton cell volume (V) was converted to carbon using the following equations: 0.288 V^0.811^ for diatoms and 0.216 V^0.939^ for non-diatoms (Menden-Deuer and Lessard, 2000).

### Statistical Analyses

Two complementary multivariate analyses were used to describe phytoplankton community structure related to environmental forcing. First, redundancy analyses (RDAs) were used to assess community variation under the constraint of environmental variables. Three different datasets were analyzed independently using this method: (i) phytoplankton community data from both surface and DCM combined to assess the main patterns of phytoplankton community structure; (ii) surface and (iii) DCM phytoplankton community data to find horizontal patterns related to the mesoscale hydrography of the two depths separately. RDAs are more appropriate than other multivariate analyses when species turnover is not very large since they assume that there is a short gradient and that the abundance of each species is likely linearly dependent on environmental variables (Ter Braak and Smilauer, 2002). A total of seven phytoplankton groups were considered: diatoms, dinoflagellates, large nanophytoplankton (5–20 μm), small nanophytoplankton (2–5 μm), picoeukaryotes, *Synechococcus* and *Prochlorococcus*. The dataset of phytoplankton groups was double-square root transformed to correct scale differences among abundances of groups. Environmental variables were selected by forward-selection by using the “forward.sel” function from “packfor” package of R. The significance and explained variation of the axes was assessed by using “rda” and “anova.cca” functions from “vegan” package of R.

Second, a combination of nonmetric multidimensional scaling (NMDS) and general additive modeling (GAM) was used to characterize potential non-linear effects between picoplankton community and environmental gradients (e.g., Muenchow et al., 2013; Hidalgo et al., 2014). The picoplankton community analyzed using this method included data from the overall photic zone considering four groups of organisms: picoeukaryotes, *Synechococcus, Prochlorococcus*, and heterotrophic prokaryotes [dataset (iv)]. The more detailed and larger picoplankton dataset enables us to assess vertical gradients and non-linear relationships. NMDS was used to summarize the relationships among samples in two unconstrained axes according to their community composition based on Bray-Curtis similarity distance matrix. GAM was used to link community structure (represented by the two first ordination axes of the NMDS for each station and depth, i.e., dimensionless values or scores) to environmental data. Spearman rank correlations were then used to relate the different picoplankton groups to the NMDS axes. A positive correlation between a specific group and the NMDS axis tentatively indicates a positive effect of the environmental variables related to the axis and vice versa. Heterotrophic picoeukaryotes were not quantified in this study, and consequently, they were not considered in the picoplankton community analyses, however, their contribution to the total heterotrophic microbes abundance is negligible compared to heterotrophic prokaryotes (Christaki et al., 2001).

The variables used for the analyses were: depth, year, temperature, salinity, AOU, ML depth, PAR, inorganic nitrogen, phosphate, silicate concentrations and Chl-*a* fluorescence. Most nitrogen concentrations were under the detection limit in surface waters, hindering the inclusion of this variable in the analyses. Chl-*a* fluorescence was used as a variable for picoplankton community analyses to relate community structure with their pigment content. Only non-colinear variables were used in models, that were selected applying the Variance Inflation Factor (VIF, Zuur et al., 2009, p. 387) analysis. ANOVA analysis was also used to identify statistical differences of abundance and biomass of taxonomic groups between surface and DCM.

## Results

### Hydrographic and Environmental Conditions

A surface salinity front, formed due to the confluence of the new and resident AW masses, was present during the 3 years sampled, although variable in location and intensity (Figure 1). In 2014 the confluence of the two water masses occurred south of the Balearic archipelago, and the new AW occupied the eastern part of the study area (Figure 1A). The front structure below the thermocline was no longer visible in the sampled area (Supplementary Figure S2A). The intrusion of new AW through the Mallorca channel in 2015 resulted in an undefined boundary with mixed new and resident AW (Figure 1B), that extended through the whole euphotic layer (Supplementary Figure S2B). An intense and well-defined front structure was observed in 2016 north of the archipelago throughout the whole euphotic layer, with a large contribution of new AW (Figure 1C and Supplementary Figure S2C). VSI was significantly correlated to surface salinity in the 3 years of study (Supplementary Figure S3), i.e., higher stratification values coincided with larger contributions of new AW (lower salinities). The front did not enhance nutrient enrichment, neither Chl-*a* concentration nor phytoplankton abundance were higher at the boundary of the two water masses.

**FIGURE 2:**
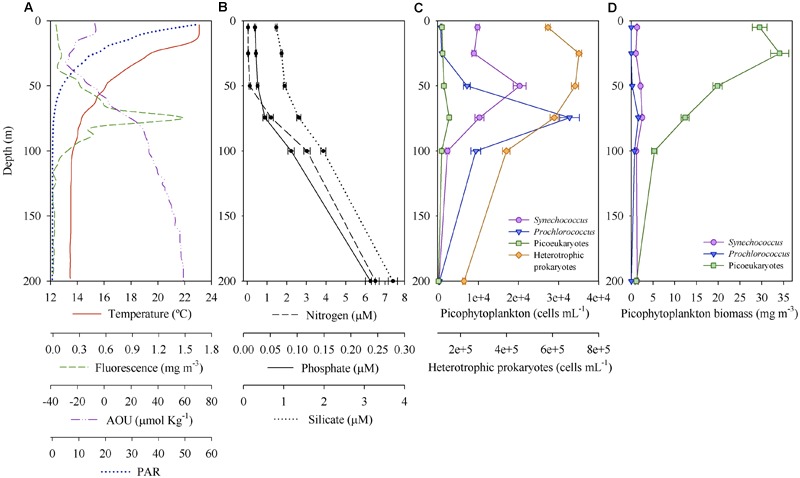
Environmental variables and picoplankton community through the photic zone. **(A)** Temperature, fluorescence, apparent oxygen utilization (AOU) and photosynthetically active radiance (PAR) at a representative station (from 2016 survey, located at the Mallorca Channel corresponding to the new Atlantic Water mass). Mean (± SE) **(B)** inorganic nutrients concentration, **(C)** picoplankton abundance and **(D)** picophytoplankton carbon biomass of the 3 years of sampling.

Figure 2A shows a representative vertical profile of temperature, fluorescence, AOU and PAR (profiles for all stations depicted in Supplementary Figure S4). The thermocline was well defined and divided the upper warmer mixed layer (average 23.7°C) from the deeper colder layer (average 14.5°C), hereafter DCM layer. The ML depth ranged between 5 and 22 m among all stations (Supplementary Figures S4, S5). The DCM was located between 50 and 100 m (Figure 2A and Supplementary Figure S4). Chlorophyll fluorescence signal was extremely low at surface (mean 0.027 ± 0.002 mg Chl-*a* m^-3^) during the 3 years, while fluorescence values at the DCM during 2014 were higher (0.86–5.28 mg m^-3^) than in 2015 (0.29–1.88 mg m^-3^) and 2016 (0.50–2.81 mg m^-3^) (Supplementary Figure S4). PAR at the DCM ranged between 0.05 and 2.87% during the 3 years sampled. AOU was always negative above 50 m, i.e., down to the thermocline, and increased gradually with depth (Figure 2A and Supplementary Figure S4). Similar vertical patterns were observed for inorganic nitrogen, phosphate and silicate, all progressively increased their concentration with depth (Figure 2B). Nitrogen concentration was under the detection limit until depths greater than 50 m for half of the samples, reaching 1.20 ± 0.22 μM at 75 m (Figure 2B). The N:P ratio was on average 5.53 ± 0.97 and 30.36 ± 1.08 above and below 50 m, respectively, for the 3 years. It is noteworthy to mention that the average surface N:P ratio is probably an overestimation, as N:P ratio was only calculated when inorganic nitrogen concentrations were above the detection limit.

### Phytoplankton Community Structure

**FIGURE 3:**
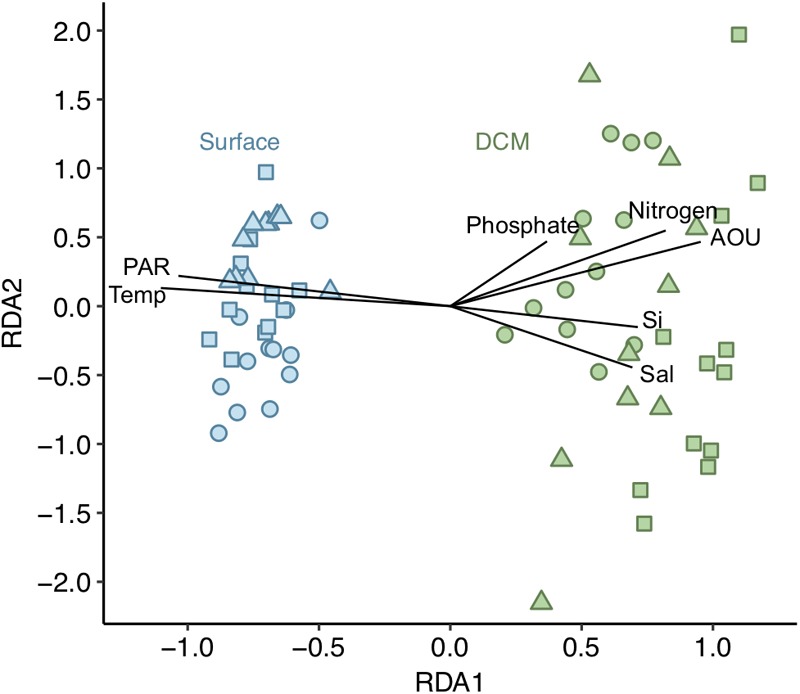
Redundancy analysis (RDA) for the phytoplankton communities [dataset (i)]. Adjusted *R*^2^ = 0.741. Phytoplankton communities from surface depicted in blue and from DCM in green. Shapes indicate the 3 years sampled: dots for 2014, squares for 2015 and triangles for 2016. Vectors represent the environmental variables selected. Length and direction of vectors indicates relative correlation strength with RDA axes. Sal, salinity; Si, silicate; temp, temperature.

The structure of the overall phytoplankton community from both surface and DCM [dataset (i)] was explored through redundancy analysis (RDA). The RDA model explained 74% of the observed variation. The first two RDA axes were significant (*p* < 0.001) and explained 89 and 10% of the variation in the model, respectively. Surface and DCM phytoplankton communities clustered separately along the first RDA axis (Figure 3). All environmental variables selected exhibited vertical gradients. The phytoplankton community was vertically stratified, with distinct community structures between the warmer and more illuminated surface layer and the DCM layer, characterized by higher AOU, salinity and nutrient concentration values (Figure 3). Diatoms, picoeukaryotes and *Prochlorococcus* were significantly more abundant at DCM than at surface during the 3 years sampled (ANOVA test, *p* < 0.05). However, dinoflagellates, nanophytoplankton and *Synechococcus* exhibited varying vertical distribution patterns and did not show significant differences between the two depth layers (ANOVA test, *p* > 0.05). *Synechococcus* dominated numerically at all stations at the upper 50 m, while *Prochlorococcus* was the dominant below 50 m (Figure 2C and Table 1).

**Table 1:** Mean (± SE) cell abundance and carbon biomass of phytoplankton groups and total phytoplankton C:Chl-*a* ratios, calculated for surface and DCM communities for the 3 years of sampling.

		2014		2015		2016	
		Surface	DCM	Surface	DCM	Surface	DCM
Abundance (cells mL^-1^)	Diatoms	0.37 ± 0.10	0.85 ± 0.12	0.44 ± 0.11	3.30 ± 1.11	0.55 ± 0.28	16.41 ± 7.38
	Dinoflagellates	0.63 ± 0.20	1.23 ± 0.60	2.40 ± 0.19	1.89 ± 0.17	1.35 ± 0.12	1.72 ± 0.14
	Large nanophytoplankton	74.26 ±3.61	105.08 ± 14.07	52.40 ± 3.00	56.54 ± 6.85	60.80 ± 5.64	71.81 ± 10.49
	Small nanophytoplankton	99.67 ± 12.47	80.52 ± 7.47	99.95 ± 9.79	107.70 ± 13.15	154.18 ± 20.82	189.38 ± 31.26
	Picoeukaryotes	1008.12 ± 48.49	2212.51 ± 423.72	872.83 ± 56.32	2545.94 ± 422.59	912.24 ± 41.31	3068.41 ± 626.10
	*Synechococcus*	13358.61 ± 1277.20	8197.79 ± 2336.25	10813.46 ± 946.88	12042.60 ± 4033.05	7441.78 ± 623.28	11481.28 ± 811.92
	*Prochlorococcus*	840.46 ± 111.25	18845.63 ± 5108.58	571.26 ± 48.40	34956.28 ± 4213.33	516.90 ± 67.90	29120.36 ± 5455.64
Biomass (mg C m^-3^)	Diatoms	0.23 ± 0.06	0.44 ± 0.07	0.21 ± 0.08	0.67 ± 0.23	0.39 ± 0.22	6.64 ± 2.23
	Dinoflagellates	1.63 ± 0.28	2.14 ± 0.66	2.79 ± 0.24	3.14 ± 0.90	3.10 ± 0.85	3.12 ± 1.04
	Large nanophytoplankton	2.88 ± 0.14	4.07 ± 0.54	2.03 ± 0.12	2.19 ± 0.26	2.36 ± 0.22	2.78 ± 0.41
	Small nanophytoplankton	0.79 ± 0.10	0.64 ± 0.06	0.79 ± 0.08	0.85 ± 0.10	1.22 ± 0.16	1.50 ± 0.25
	Picoeukaryotes	38.16 ± 7.64	16.96 ± 3.00	34.40 ± 3.54	10.71 ± 2.05	22.68 ± 2.30	13.77 ± 2.66
	*Synechococcus*	1.74 ± 0.20	2.23 ± 0.36	1.33 ± 0.13	2.49 ± 0.63	0.97 ± 0.09	3.17 ± 0.45
	*Prochlorococcus*	0.04 ± 0.01	1.23 ± 0.33	0.02 ± 0.00	1.79 ± 0.23	0.01 ± 0.00	1.48 ± 0.31
C:Chl-*a* ratio	Total	–	43.88 ± 5.42	651.11 ± 46.14	42.36 ± 3.01	327.19 ± 31.56	29.92 ± 2.18
	Microphytoplankton	–	–	-	-	305.05 ± 121.64	65.04 ± 23.70
	Nanophytoplankton					154.52 ± 19.43	20.22 ± 3.43
	Picophytoplankton					405.06 ± 48.23	34.27 ± 3.69

*Size-fractionated C:Chl-a ratios in 2016 for both depths are also indicated. Surface Chl-a data was not collected in 2014, hence C:Chl-a ratio is not available.*

**FIGURE 4:**
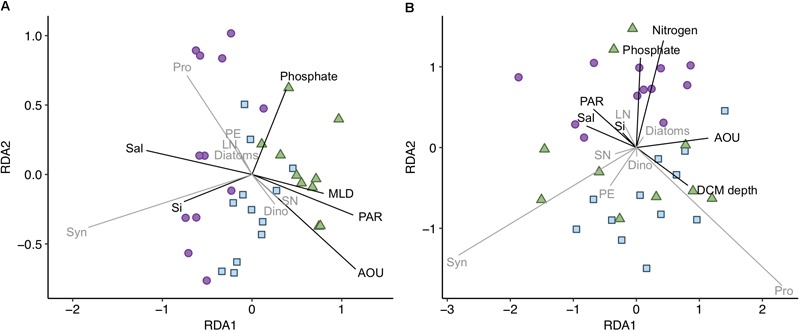
Redundancy analysis (RDA) for the **(A)** surface and **(B)** DCM phytoplankton communities [dataset (ii) and (iii), respectively]. Adjusted *R*^2^ = 0.311 and 0.475 for **(A,B)**, respectively. Shapes and colors indicate the 3 years sampled: purple dots for 2014, blue squares for 2015 and green triangles for 2016. Vectors represent the environmental variables (black vectors) and phytoplankton groups (gray vectors) constrained in the RDA model. Length and direction of vectors indicates relative correlation strength with RDA axes. MLD, mixed layer depth; Sal, salinity; Si, silicate; Pro, *Prochlorococcus*; Syn, *Synechococcus*; PE, picoeukaryotes; SN, small nanophytoplankton; LN, large nanophytoplankton; Dino, dinoflagellates.

The community composition was additionally analyzed for surface and DCM separately [datasets (ii) and (iii), respectively]. At both depths there were significant differences in phytoplankton community structure across years, in particular 2014 communities clearly differed from 2015 and 2016 communities (Figure 4A,B). Surface RDA model (31% variation explained) showed that surface waters in 2014 had higher salinity and silicate concentration and lower AOU, PAR, and ML depth values than during the latter 2 years (Figure 4A and Supplementary Figure S4), explained by the first RDA axis (72% of the model variation; *p* < 0.01). Higher salinity in 2014 is related to a larger contribution of resident AW mass in this layer (Figure 1A), suggesting different phytoplankton community structure in the two water masses distinguished, i.e., new and resident AW. *Synechococcus* and *Prochlorococcus* were more abundant in 2014 at higher salinity and silicate concentration as compared to 2015 and 2016, while dinoflagellates and small nanophytoplankton were more abundant in 2015 and 2016 at lower salinity and higher AOU, PAR, and ML depth values as compared to 2014 (Figure 4A).

DCM RDA model (47% variation explained) showed that light and nutrient availability influenced community structure. The first two RDA axes explained 66 and 25% of the variation in the model. The DCM phytoplankton communities were influenced by the depth of the DCM, related to PAR and AOU values (Figure 4B). *Prochlorococcus* was associated to deeper DCMs and lower irradiances, corresponding to strongly stratified stations with lower salinities (i.e., larger new AW mass contribution) (Figure 4B and Supplementary Figure S2). The year-associated difference at the DCM is shown along the second axis of the RDA (Figure 4B). The DCM in 2014 presented higher nutrient concentrations and was related to higher diatoms and large nanophytoplankton and lower picophytoplankton and dinoflagellates abundances as compared to 2015 and 2016 (Figure 4B).

**FIGURE 5:**
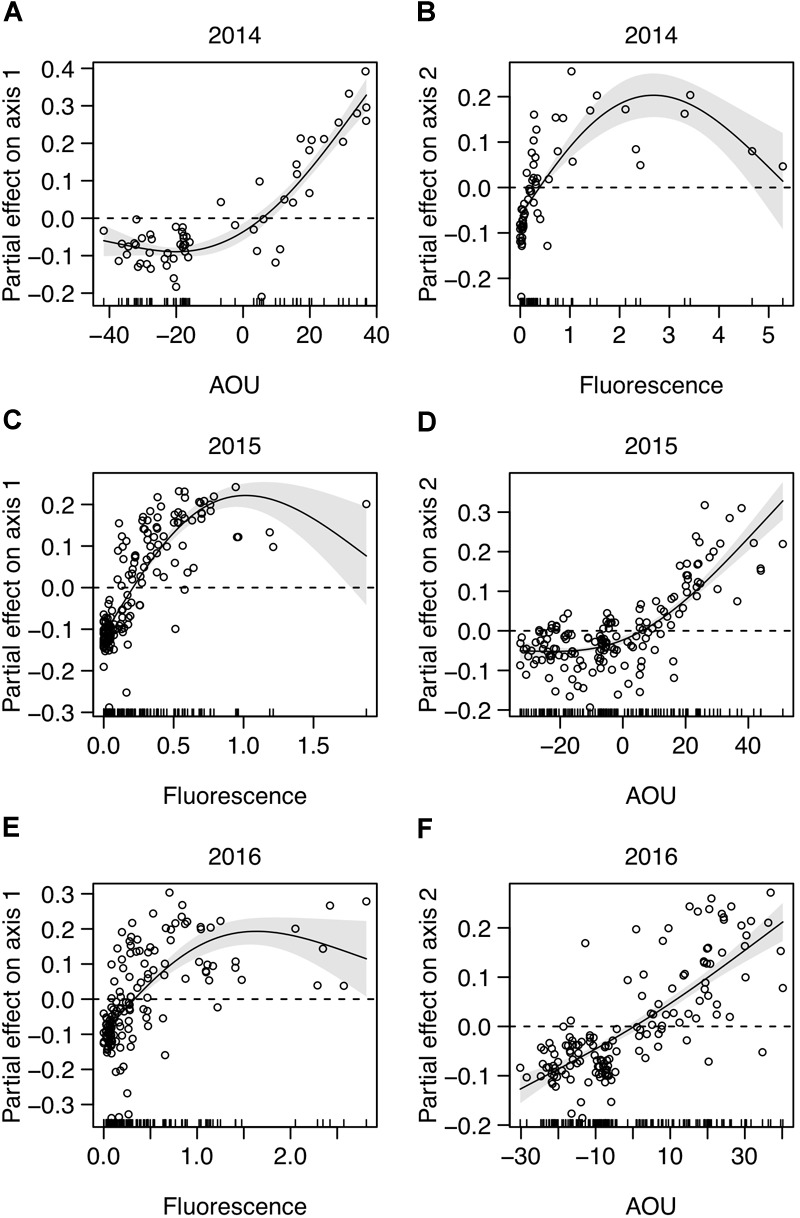
Environmental factors related to picoplankton community structure from the generalized additive models (GAM) (Table 2). Plots show the significant partial effect of the variable on **(A,C,E)** Axis 1 and **(B,D,F)** Axis 2 of NMDS analyses [dataset (iv)], computed for each year. Positive values indicate a positive effect of the variable on the NMDS Axis; negative values indicate a negative effect. Fitted regression line (solid), 95% confidence intervals (gray areas) and partial residuals are shown. NMDS plots and residual plots for each GAM model in Supplementary Figures S6, S7, respectively.

The picoplankton community structure [dataset (iv), including picophytoplankton and heterotrophic prokaryotes] was further analyzed by NMDS ordination (NMDS plots in Supplementary Figure S6) and GAM regressions. The overall results show that the picoplankton community structure was essentially related to fluorescence and AOU (Table 2). Note that these environmental variables were related to either Axis 1 or 2 depending on the year (Table 2). The GAM modeling indicates that the communities with higher fluorescence values, corresponding to the DCM depth, were significantly different from the communities with lower fluorescence values (i.e., above and below the DCM) (Figure 5B,C,E). Moreover, the results show a non-linear saturation effect of fluorescence on the NMDS Axes (on Axis 1 in 2015 and 2016 and on Axis 2 in 2014; Table 2 and Figure 5B,C,E). The groups positively correlated (picoeukaryotes and *Prochlorococcus* at all years, Table 3) were associated to the highest fluorescence values, in agreement with their dominance in the DCM. The spatial structure of picoplankton was also influenced by AOU (Axis 1 in 2014 and Axis 2 in 2015 and 2016; Table 2). Communities inhabiting waters with positive AOU values, i.e., waters below the thermocline, were significantly different from communities in negative AOU waters, i.e., thermocline and waters above it (Figure 5A,D,F). Besides, the results also show that for positive AOU values there was a positive linear relation with the NMDS axes (Figure 5A,D,F). Thus, picoplankton groups that negatively correlated to the NMDS axes (Axis 1 in 2014 and Axis 2 in 2015 and 2016; Table 3) were associated to negative AOU values, corresponding to surface waters until the base of the thermocline.

**Table 2:** Results of the generalized additive models (GAM) for the first and second axes of the nonmetric multidimensional scaling (NMDS) analyses computed for each sampling year on the picoplankton community [dataset (iv)].

Year	Variable	df	*p*-value	%DE	*R*^2^	n
**GAM model – Axis 1 NMDS**			
2014	*AOU*	1.97	<0.0001	82.0	0.81	60
2015	*Fluorescence*	1.98	<0.0001	71.2	0.71	157
2016	*Fluorescence*	1.96	<0.0001	44.2	0.43	144
**GAM model – Axis 2 NMDS**			
2014	*Fluorescence*	1.97	<0.0001	56.0	0.54	60
2015	*AOU*	1.97	<0.0001	62.7	0.62	157
2016	*AOU*	1.58	<0.0001	59.9	0.59	144

*The environmental variable explaining most of the variation, the estimated degrees of freedom (df), the probability (p-value), the deviance explained (%DE), the R^2^ and the sample size (n) are included for each model.*

**Table 3:** Spearman rank correlation coefficient between picoplankton group abundances and ordination axes of the nonmetric multidimensional scaling (NMDS) analyses for the picoplankton community [dataset (iv)].

	2014	2015	2016
**Axis 1 NMDS**		
Picoeukaryotes	-0.609^∗∗∗^	0.750^∗∗∗^	0.679^∗∗∗^
*Synechococcus*	-0.958^∗∗∗^	–	0.561^∗∗∗^
*Prochlorococcus*	–	0.961^∗∗∗^	0.865^∗∗∗^
Heterotrophic prokaryotes	-0.793^∗∗∗^	–	0.327^∗∗∗^
**Axis 2 NMDS**		
Picoeukaryotes	0.469^∗∗∗^	-0.189^∗^	–
*Synechococcus*	–	-0.969^∗∗∗^	-0.614^∗∗∗^
*Prochlorococcus*	0.981^∗∗∗^	–	-0.576^∗∗∗^
Heterotrophic prokaryotes	–	-0.766^∗∗∗^	-0.550^∗∗∗^

*All analyses were computed for the three sampled years separately and only significant correlations (p < 0.05) are presented. Number of asterisks indicate the significance level of the correlations (^∗∗∗^p < 0.0001, ^∗^p < 0.05). Dashes indicate non-significant correlations.*

### Phytoplankton Carbon Biomass and Chlorophyll-*a*

**FIGURE 6:**
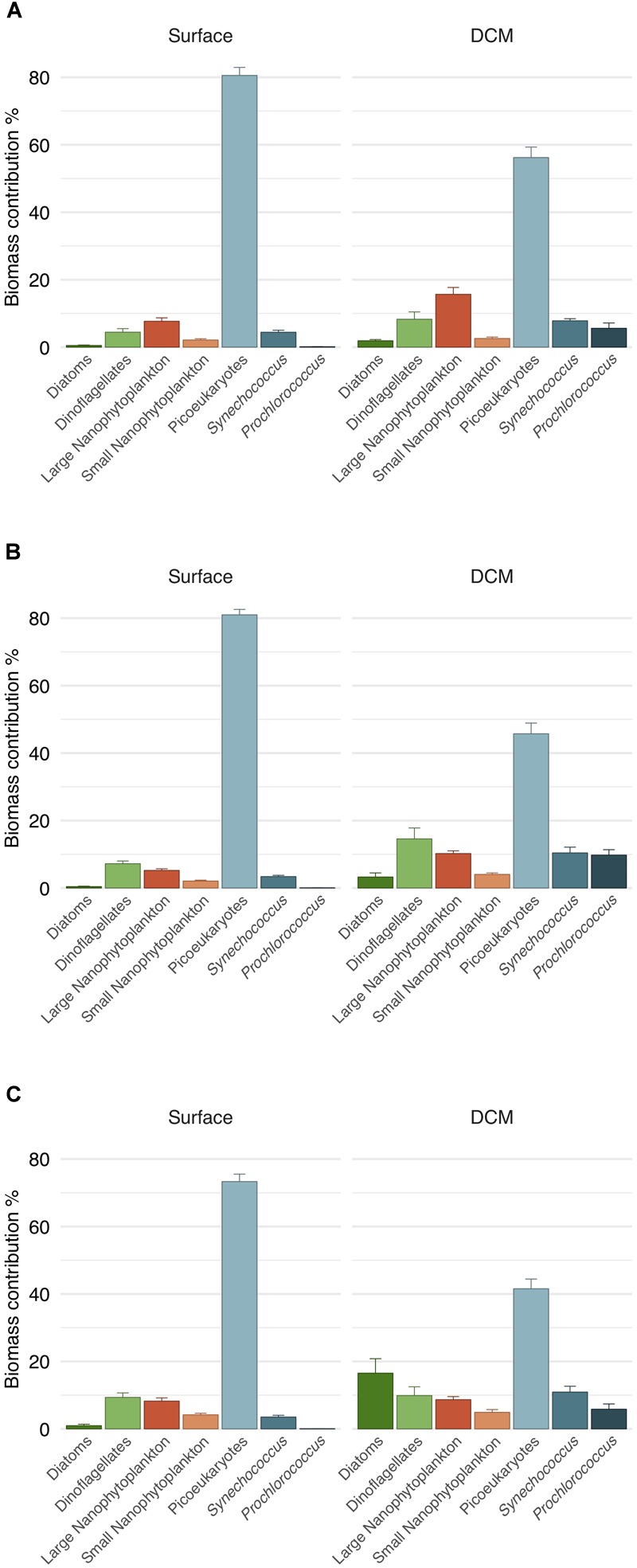
Carbon biomass contribution of phytoplankton groups to total phytoplankton biomass. Mean values calculated for surface and DCM for **(A)** 2014, **(B)** 2015, and **(C)** 2016. Vertical lines indicate the standard error.

The carbon biomass was dominated by the picophytoplankton fraction (82.22 ± 1.21% and 64.62 ± 2.05% of total community biomass at surface and DCM, respectively). In particular, picoeukaryotes were the major contributors to phytoplankton biomass, both in surface (70–80%) and in DCM (40–55%) (Figure 6). All groups with the exception of picoeukaryotes increased their relative contribution to carbon biomass at DCM (Figure 6). Noteworthy, the contribution of diatoms biomass increased at DCM in 2016 (16% of total biomass) due to elevated diatom density (about 70 cells mL^-1^) at two stations that year (Table 1 and Figure 6C). *Prochlorococcus* exhibited significantly higher carbon biomass at DCM than at surface throughout the 3 years sampled (ANOVA test, *p* < 0.01); whereas picoeukaryotes exhibited significantly higher carbon biomass at surface than at DCM (ANOVA test, *p* < 0.05). The vertical distribution of carbon biomass of the three picophytoplankton groups indicated the large contribution of picoeukaryotes, especially in the first 25 m of the water column (Figure 2D). Dinoflagellates and small nanophytoplankton biomass were not significantly different between depths (ANOVA test, *p* > 0.1). Diatoms, large nanophytoplankton and *Synechococcus* biomass exhibited significant differences depending on the year. Significantly higher carbon biomass at DCM in 2014 and 2015 for diatoms, in 2014 for large nanophytoplankton and in 2016 for *Synechococcus* (ANOVA tests, *p* < 0.05). Average total phytoplankton carbon biomass for the euphotic water layer was similar the 3 years: 36.98 ± 4.56, 32.45 ± 3.06, and 31.90 ± 2,84 mg C m^-3^ in 2014, 2015, and 2016, respectively. However, surface total biomass was twofold higher in 2015 as compared to DCM (ANOVA test, *p* < 0.001), due to an increase in the picoeukaryotic biomass at surface. No significant differences in total carbon biomass were found between surface and DCM in 2014 and 2016 (ANOVA test, *p* > 0.05).

We calculated total C:Chl-*a* ratio for each year and both depths (Table 1). The C:Chl-*a* ratio was remarkably higher at surface than at DCM, due to more than eightfold Chl-*a* concentration at DCM as compared to surface (Table 1). The C:Chl-*a* ratio in 2016 at surface and DCM was lower than in 2014 and 2015, due to relatively higher Chl-*a* concentrations in 2016. Size-fractionated C:Chl-*a* ratios were also calculated for the 2016 survey (Table 1). The C:Chl-*a* ratio of picophytoplankton was larger than that of nanophytoplankton at surface; whereas, the C:Chl-*a* ratio of microphytoplankton was not significantly different from pico- or nanophytoplankton. C:Chl-*a* ratios at DCM were different between the three phytoplankton size groups, with maxima for microphytoplankton and minima for nanophytoplankton fraction (Table 1).

### Change of Picophytoplankton Cell Volume With Depth

**FIGURE 7:**
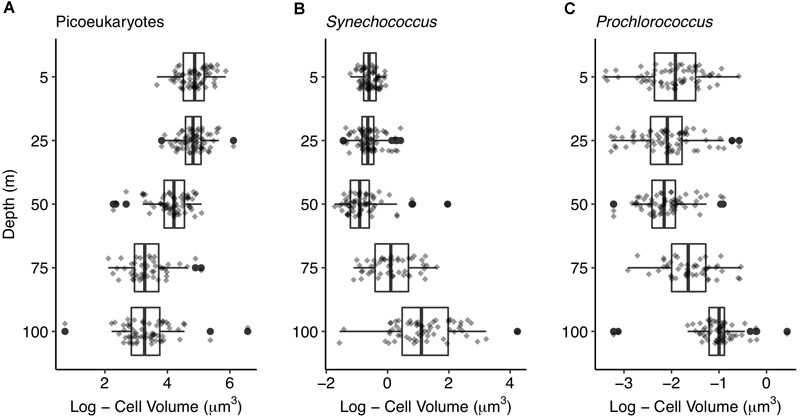
Picophytoplankton size change with depth. Boxplots showing cell volumes at each depth for **(A)** picoeukaryotes, **(B)** *Synechococcus*, and **(C)** *Prochlorococcus*. Dark vertical lines inside the boxes indicate median, boxes indicate the first and third quartile and extending lines indicate the variability outside the first and third quartile. Outliers (black dots) and jitter values (gray diamonds) are shown.

Cell volume was significantly related to depth for the three picophytoplankton groups. Picoeukaryotic cells were larger at surface with decreasing cell volume down to 100 m depth (Figure 7A). Contrarily, the largest *Synechococcus* and *Prochlorococcus* cells were observed at deeper layers. Prokaryotes cell volume did not show significant differences above 50 m depth (i.e., down to the thermocline), increasing significantly below 50 m depth (Figure 7B,C). Micro- and nanophytoplankton did not show significant cell size differences between surface and DCM communities (data not shown).

## Discussion

The expansion of ocean’s oligotrophic regions and the increase of water column stratification are two of the main predicted climate change consequences to which phytoplankton communities will be exposed to (Polovina et al., 2008; Capotondi et al., 2012). We identified the patterns of the phytoplankton community composition, structure and biomass under oligotrophy and summer stratification conditions across the euphotic zone of the western Mediterranean Sea in three consecutive years. The strength of the thermocline separated two contrasting environments with different resource limitation (nutrients versus light) that modulated the structure of phytoplankton community. Environmental conditions in the study area were typical of oligotrophic and stratified regions (D’Ortenzio and Ribera d’Alcalà, 2009; Powley et al., 2017), with extremely low nutrient and Chl-*a* concentrations at surface and a shallow ML depth, characteristic of the early summer in the Mediterranean Sea (Pasqueron de Fommervault et al., 2015a).

In this study, the phytoplankton community composition was strongly influenced by the water column stratification, in agreement with previous studies (Pérez et al., 2006; Ramfos et al., 2006; Bouman et al., 2011; Hidalgo et al., 2014; Mojica et al., 2015). The picoplankton fraction dominated in terms of abundance and biomass at all stations sampled and within all the photic zone, congruent with the oligotrophic conditions found (Mojica et al., 2015; Cotti-Rausch et al., 2016; Otero-Ferrer et al., 2018). Under nutrient-limited conditions, smaller cells have an advantage for nutrient uptake as compared to larger cells (Marañón, 2015). Diatoms, with relatively larger cell size, were negligible at surface and always significantly more abundant at the DCM layer, related to the higher nutrient supply at this depth as compared to surface. The fact that dinoflagellates and nanophytoplankton (i.e., larger sizes) did not tend to increase with nutrient availability suggests that these groups may endure better nutrient-limiting conditions than diatoms, in agreement with the general knowledge of the ecology of these groups (Reynolds, 2006). The dominance of dinoflagellates and nanophytoplankton under nutrient limiting conditions has also been related to the potential for a mixotrophic lifestyle (i.e., combination of autotrophy and heterotrophy) described in some species of dinoflagellates and nanophytoplankters (Unrein et al., 2007; Siokou-Frangou et al., 2010; Unrein et al., 2013). This strategy might be particularly advantageous in oligotrophic environments where it can supplement C fixation (Stoecker et al., 2017). It should be noted that the nanophytoplankton abundances reported in this study are low (2.5 times lower) compared to other studies from the open Mediterranean Sea (Di Poi et al., 2013). This result might be indicative of an underestimation of the nanophytoplanktonic fraction enumerated using light microscopy as compared to epifluorescence microscopy.

The overall results show that stratification and vertical nutrient and light gradients are the main drivers of the phytoplankton community. Within the photic layer, stratification causes the sharp gradient in nutrient availability and N:P ratio above and below the thermocline. Below the thermocline, nutrient and light availability is strongly modulating the composition of the communities (Latasa et al., 2017). The components of the phytoplankton community have shown differences in nutrient uptake efficiency and requirements (Agawin et al., 2004; Latasa et al., 2010; Mouriño-Carballido et al., 2016) and in light quantity and quality adaptation (Bouman et al., 2006; Mella-Flores et al., 2012). Although N:P ratio results should be treated with caution due to the limited number of surface nitrogen concentrations above detection limit, the vertical patterns of N:P ratios in this study suggest differences in the nutrient limitation above and below the thermocline. The N:P ratio at the surface layer (N:P < 6) indicates nitrogen-limitation while below the thermocline (N:P > 30) suggests phosphorous-limitation. A general phosphorous-limitation during the summer stratification period has been described for the Mediterranean Sea (Marty et al., 2002; Pasqueron de Fommervault et al., 2015b; Powley et al., 2017). On the other hand, the western basin is influenced by the surface and nitrogen-limited AW inflow (Lazzari et al., 2016; Powley et al., 2017), which supports the N:P ratios reported in this study. These differences in nutrient composition should be considered when assessing the vertical patterns of distribution of phytoplankton groups. In agreement with results from previous studies from the south-west Mediterranean basin, the vertical abundance patterns of *Synechococcus* and *Prochlorococcus* seems to be also influenced by the different sensitivity to light stress (Mella-Flores et al., 2012). Furthermore, the low abundance of *Prochlorococcus* at surface coincides with the reported scarcity of high-light (HL) adapted populations in the southern Mediterranean (Mella-Flores et al., 2011; Mena et al., 2016).

The picoplankton community at the DCM was significantly different from the communities from other depths throughout the photic zone, regardless of the Chl-*a* fluorescence concentration at the DCM. The differentiation of the DCM community reflects the niche adaptation of autotrophs based on the balance between light level and nutrient availability (Estrada et al., 2016; Latasa et al., 2017). Besides, AOU was also related to the picoplankton community structure within the water column. The picoplankton communities in the surface over-oxygenated zone (negative AOU values, i.e., oxygen concentration above saturation) were significantly different from the communities located in the under-saturated oxygen zone below the thermocline (positive AOU values). Below the thermocline, the relationship between AOU and community structure involves the increased heterotrophic activity and oxygen consumption rates (Williams et al., 2004). The relationship between DCM phytoplankton community structure and the depth of the DCM was driven by light availability. The deepening of the DCM is linked to lower irradiances which favored *Prochlorococcus*. Different light-adapted ecotypes inhabiting different niches along the euphotic zone have been described for *Prochlorococcus* (West and Scanlan, 1999; Mella-Flores et al., 2011), which suggest the prevalence of low-light (LL) adapted populations in this area. Unfortunately, the abundance of *Prochlorococcus* ecotypes was not assessed during this study. Contrary to the surface phytoplankton, the DCM phytoplankton communities did not significantly differ between the two AW masses. However, we cannot exclude an effect of the mesoscale dynamics since the front structure at the DCM depth was only well-defined in the last survey. Sub-mesoscale processes (Cotti-Rausch et al., 2016; Pascual et al., 2017) and vertical diffusivity (Mouriño-Carballido et al., 2016) are also mechanisms likely influencing phytoplankton communities, though not observed with the spatial resolution of this study.

The low total phytoplankton biomass observed in our study is similar to the phytoplankton biomass observed in a nearby station under summer stratification (Pedrós-Alió et al., 1999), and supports the oligotrophic conditions of the area. Although phytoplankton composition and structure was clearly stratified, total phytoplankton biomass at surface was similar to the DCM or even higher, in agreement with previous studies reporting that the DCM does not necessarily correspond to the biomass maximum (Pérez et al., 2006 and references therein).

Picoeukaryotes largely dominated the phytoplankton biomass throughout the photic zone, especially at surface (up to 80% of total phytoplankton biomass). The higher biovolume of picoeukaryotes results in the higher biomass contribution of this group to the community as compared to their prokaryotic counterparts (Worden et al., 2004; Grob et al., 2007; Massana, 2011). The increase in cell size of picoeukaryotes at surface and the low Chl-*a* concentration results in the elevated C:Chl-*a* ratios of the picophytoplankton fraction at this depth. Cell size increase has been suggested to be caused by limited cell division under nutrient starvation (Mouriño-Carballido et al., 2016). However, it should be noted that different methodologies were used to estimate biovolumes, and different conversion factors were used to estimate carbon content for the different phytoplankton size fractions. Moreover, despite the lower abundance as compared to prokaryotic picophytoplankton, picoeukaryotes exhibit high growth rates and can account for a large fraction of the primary production (Li, 1995; Worden et al., 2004; Jardillier et al., 2010). Fawcett et al. (2011) evidenced the large contribution of picoeukaryotes to biomass downwards export during summer stratified conditions and their role in the assimilation of newly upwelled nitrogen sources. Contrastingly, prokaryotic phytoplankton would rely on recycled nitrogen within the surface layer. Considering that picoeukaryotes were the main contributors to carbon biomass during the summer stratification period in the Mediterranean, further studies on their contribution to the primary production and on their channeling to higher trophic levels in the open Mediterranean Sea (e.g., by assessing grazing rates) will shed light on their role in the marine food webs (Worden and Not, 2008) and the carbon fluxes in oligotrophic stratified marine ecosystems.

Though a similar vertical pattern in community composition was observed for all years, interannual variation in phytoplankton community structure was apparent at both surface and DCM. Particularly, the 2014 phytoplankton community was significantly different than in 2015 and 2016. The different phytoplankton community observed in 2014 coincides with the different location of the salinity front at surface during this year, i.e., south of the archipelago. Balbín et al. (2014) revealed that the northward drift of the new AW mass through the island channels, i.e., the position of the front, depends on occurrence or absence of winter intermediate water formation events. Besides, the seasonal surface water heating was found later in 2014 as compared to the following year. These results suggest that the interannual variability in environmental conditions and physical forcing (winter mixing, water mass formation, atmospheric forcing, …) can also disrupt the structure of phytoplankton communities (Bosc et al., 2004; Pannard et al., 2008; Arin et al., 2013; Estrada et al., 2014). At the DCM the interannual variation was observed due to differences in nutrient concentration, the higher nutrient levels in 2014 resulted in higher Chl-*a* levels and diatom abundance and lower picophytoplankton abundance compared to 2015 and 2016. However, a large variation in both surface (69%) and DCM (52%) RDA models remains unexplained, suggesting that the horizontal patters could not be only explained by bottom-up control and might be highly influenced by other drivers. Studies on vertical zooplankton distribution showed that vertical stratification can hinder the migration of some small zooplankton groups and suggest different grazing pressures above and below the thermocline (Norrbin et al., 1996; Miloslavić et al., 2015). Considering that zooplankton filter feeders and heterotrophic flagellates are the main grazers of the predominant picophytoplankton during summer stratification (Calbet et al., 2008; Guo et al., 2014), further research should focus on whether differences in phytoplankton grazing pressure, associated to the different predators and phytoplankton communities composition, exists between the two water masses.

## Conclusion

This study provides evidence of the phytoplankton community composition and size structure variability at a regional scale and reveals interannual fluctuations. Overall, our results show that the phytoplankton community structure is stratified, mainly modulated by vertical gradients in different environmental factors. The thermocline differentiates two contrasting environments, characterized by different nutrient availability. The surface, characterized by limiting nutrient conditions, supports a community dominated by picophytoplankters. Below the thermocline the phytoplankton community is driven by light availability. The DCM layer and its depth involves a differentiation of the community structure associated to different capabilities of phytoplankton groups to cope with light limitation. *Prochlorococcus* is better adapted to deeper and light-limited DCM. Additionally, mesoscale dynamics, such as front structures, and interannual variability have an effect in the phytoplankton community. We provide evidence of the relevant contribution of picoeukaryotes in the stratified oligotrophic Mediterranean Sea, up to 80% of total phytoplankton biomass at surface, suggesting they might play a key role as carbon drivers in the ecosystem. Further research should focus on the potential role of mixotrophic activity and the vertical patterns between new and recycled production (Selmer et al., 1993; Estrada, 1996; Pedrós-Alió et al., 1999; Diaz and Raimbault, 2000). Understanding the role of these organisms and their impact in the biological carbon pump is essential in the current framework of increasing stratification and oligotrophic conditions in the ocean with climate change.

## Data Availability

The datasets generated for this study are available on request to the corresponding author.

## Author Contributions

CM and PR conceived and planned the work and wrote the first draft of the manuscript. CM, RS, MM, and ES carried out the sampling and laboratory work. RB contributed to physical oceanography data and interpretation. CM analyzed the data. MH contributed to the statistical analyses. All authors discussed, contributed to the final version of the manuscript, and approved the submitted version of manuscript.

## Conflict of Interest Statement

The authors declare that the research was conducted in the absence of any commercial or financial relationships that could be construed as a potential conflict of interest.
