# Supplementary material for: Phytoplankton Community Structure Is Driven by Stratification in the Oligotrophic Mediterranean Sea
Source: Front Microbiol. 2019 Jul 24;10:1698. doi: 10.3389/fmicb.2019.01698 (PMC6667633; doi:10.3389/fmicb.2019.01698)
Supplement: Supplementary file 1 [file Table_1.docx]

Supplementary Material

Phytoplankton Community Structure is Driven by Stratification in the Oligotrophic Mediterranean Sea

Catalina Mena*, Patricia Reglero, Manuel Hidalgo, Eva Sintes, Rocío Santiago, Melissa Martín, Gabriel Moyà, Rosa Balbín

*** Correspondence:** c.mena.oliver@gmail.com

# Supplementary Figures

**Supplementary Figure S1.** Representative flow cytometry cytograms of the natural picoplankton community discriminating the different groups: (A) side scatter (SSC) versus red fluorescence to differentiate the three picophytoplankton groups (*Prochlorococcus, Synechococcus* and picoeukaryotes), (B) orange fluorescence versus red fluorescence to better discriminate *Synechococcus* and picoeukaryotes based on phycoerythrin fluorescence of the formers, and (C) SSC versus green fluorescence to enumerate total prokaryotes, previously stained with SYBR green I. Fluorescence beads were added to all samples as an internal standard. The drawn polygons outline the gating for each picoplankton group. The three cytograms derived from different samples taken at the DCM depth during summer.


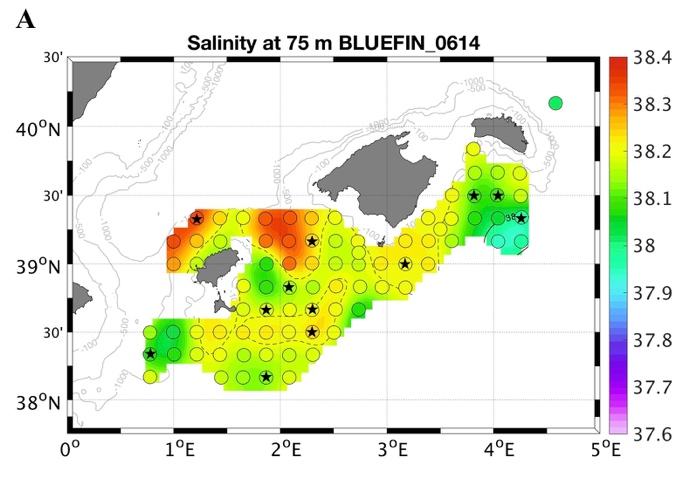

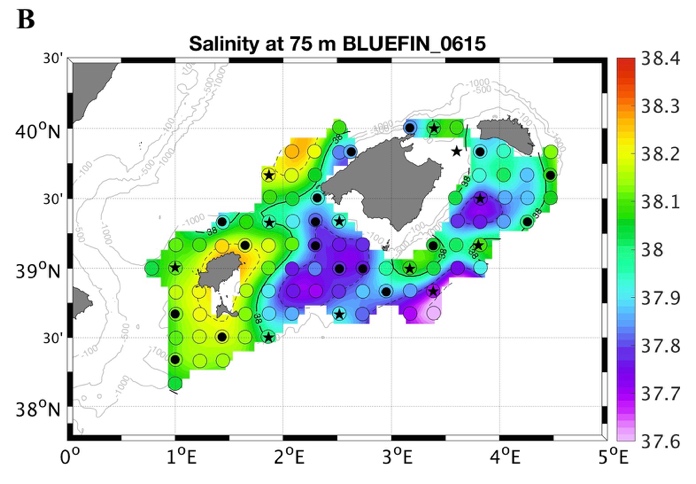

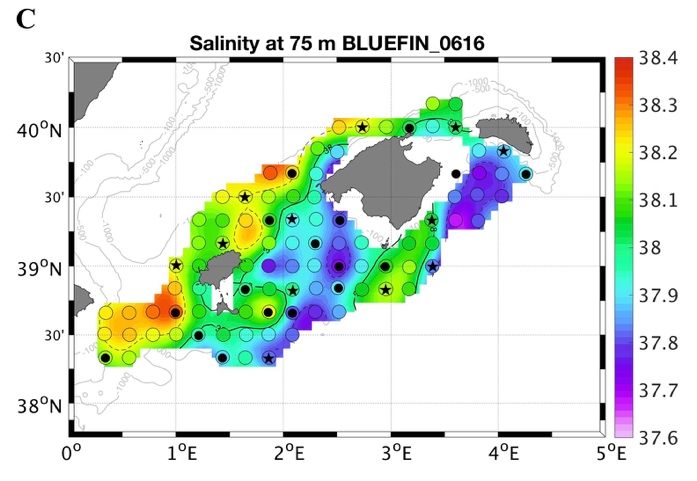


**Supplementary Figure S2.** Salinity front at the DCM layer. Interpolated maps of salinity at 75 m representative of the DCM layer for (A) 2014, (B) 2015 and (C) 2016. Values used from every CTD station from a systematic survey design in open circles (Alemany et al., 2010). Black stars and dots indicate the stations where total phyto- and picoplankton were sampled, respectively.


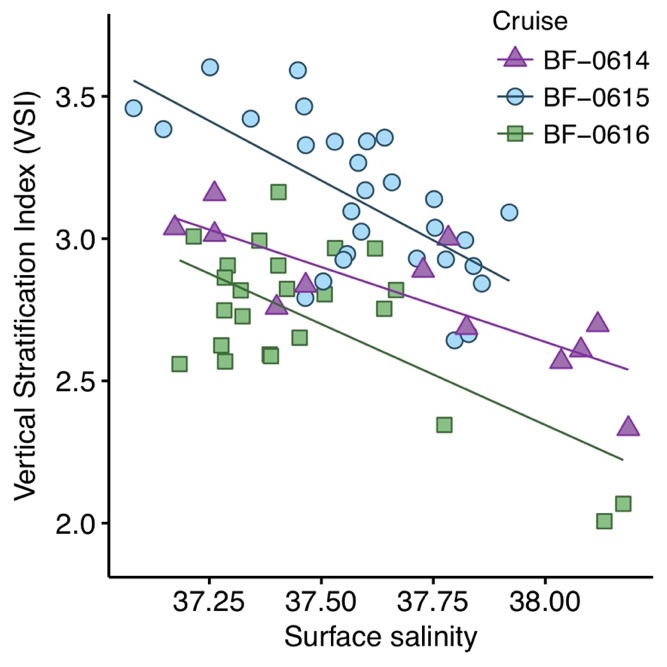


**Supplementary Figure S3.** Relationship between surface salinity and vertical stratification index (VSI) for the three cruises. The slopes from linear regressions were significant for the three years: p = 0.001, R^2^ = 0.67 for BF-0614 (2014); p < 0.001, R^2^ = 0.43 for BF-0615 (2015); p < 0.001, R^2^ = 0.43 for BF0616 (2016). The VSI was not significantly correlated to day of year or surface temperature for the three years (p values = 0.1, R^2^ < 0.2).


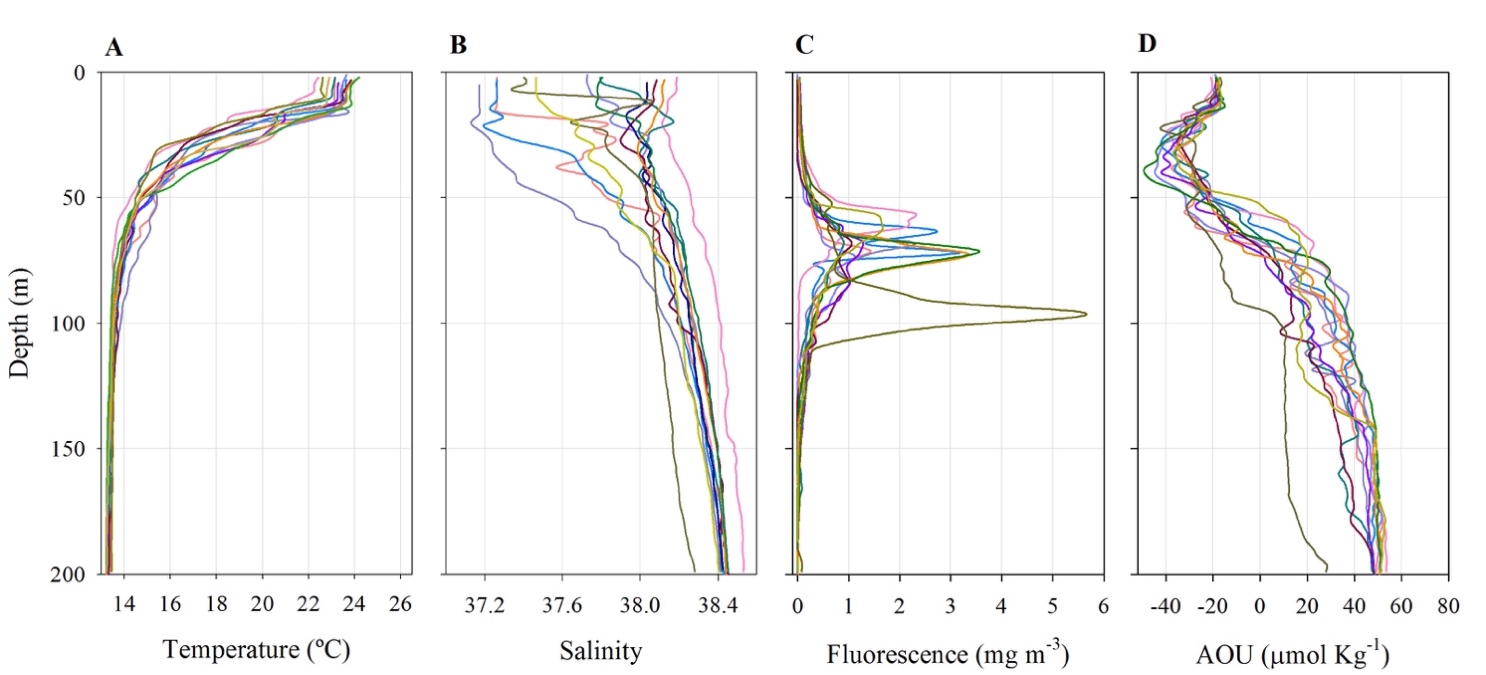


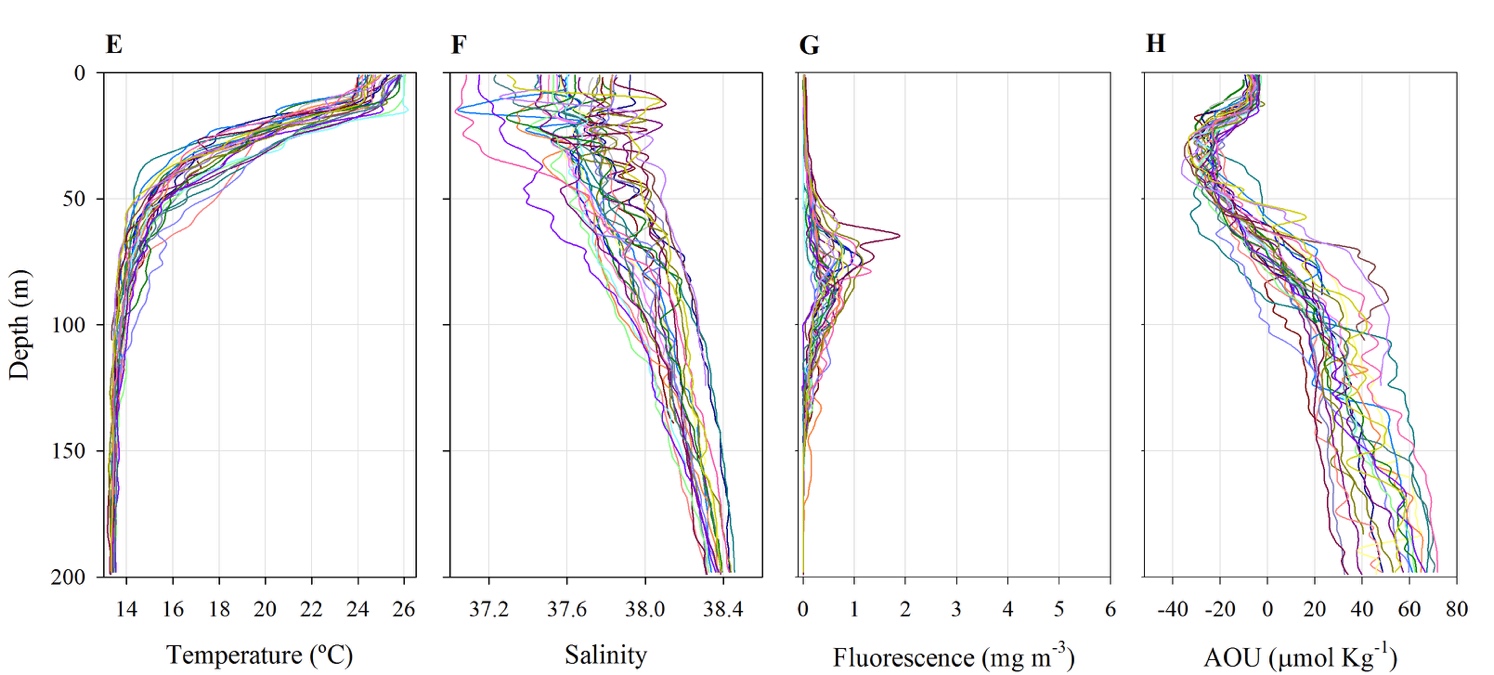

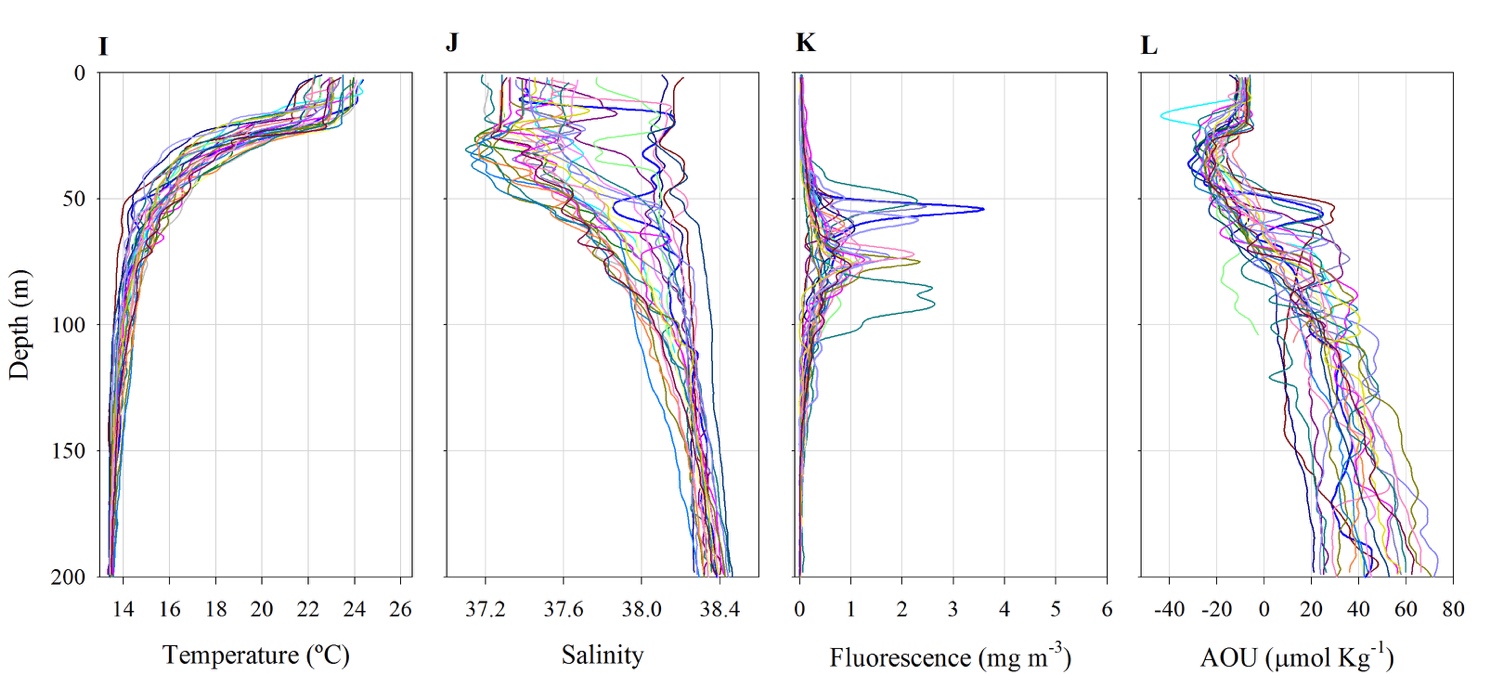


**Supplementary Figure S4.** Environmental variables throughout the photic zone. Vertical profiles of temperature, salinity, fluorescence and AOU at all stations sampled in (A-D) 2014, (E-H) 2015 and (I-L) 2016 cruises.


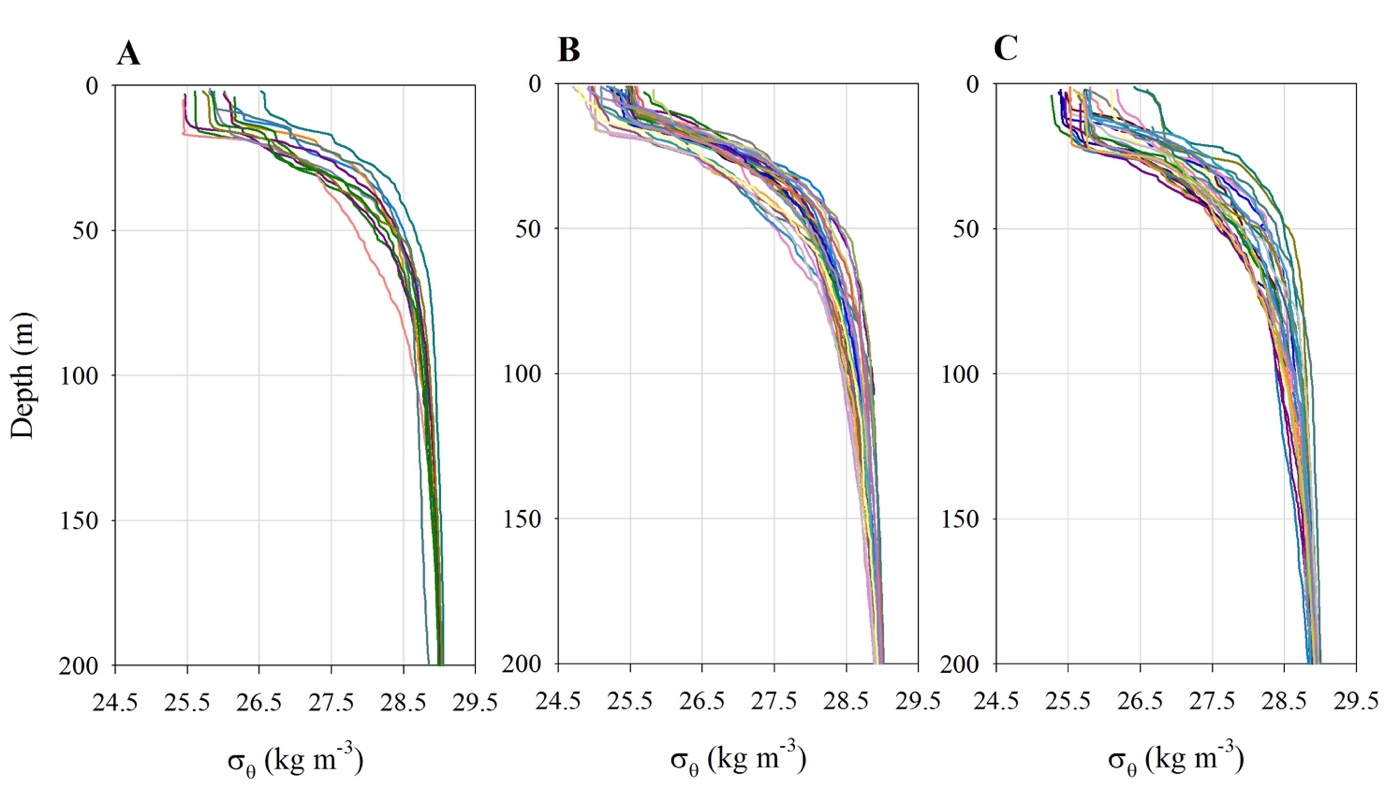


**Supplementary Figure S5.** Vertical potential density anomaly (kg m^-3^) profiles for all stations sampled in (A) 2014, (B) 2015 and (C) 2016.

**Supplementary Figure S6.** Non-metric multidimensional scaling (NMDS) plots of the first and second axis of the picoplankton community analyses (dataset [iv]) for (A) 2014, (B) 2015 and (C) 2016. The stress value indicates the quality of the ordination for each NMDS.

**Supplementary Figure S7.** Residual plots for each generalized additive model (GAM) of the picoplankton community analyses (GAM plots and model results shown in Figure 5 and Table 2, respectively). Panels A, C, E for GAM models with Axis 1 of NMDS for year 2014, 2015 and 2016, respectively. Panels B, D, F for GAM models with Axis 2 of NMDS for year 2014, 2015 and 2016, respectively. For each panel, normal Q-Q plot (upper left), plot of residuals against the linear predictor (upper right), histogram of residuals (lower left) and plot of response against fitted values (lower right) are shown. The ‘gam.check’ function from ‘mgcv’ package of R software was used for the analyses.
